# Development and evaluation of a monoclonal antibody-based competitive ELISA for the detection of antibodies against H7 avian influenza virus

**DOI:** 10.1186/s12917-021-02772-6

**Published:** 2021-02-02

**Authors:** Yuan Li, Hongliu Ye, Meng Liu, Suquan Song, Jin Chen, Wangkun Cheng, Liping Yan

**Affiliations:** 1 MOE Joint International Research Laboratory of Animal Health and Food Safety, Jiangsu Engineering Laboratory of Animal Immunology, Jiangsu Detection Center of Terrestrial Wildlife Disease, Institute of Immunology and College of Veterinary Medicine, Nanjing Agricultural University, Nanjing, Jiangsu 210095 People’s Republic of China; 2 Institute of Veterinary Immunology & Engineering, Jiangsu Academy of Agricultural Sciences, Nanjing, Jiangsu 210014 People’s Republic of China; 3 Nanjing Hongshan Forest Zoo, Nanjing, Jiangsu 210000 People’s Republic of China

**Keywords:** Avian influenza virus, H7 subtype, Competitive ELISA, Antibody detection

## Abstract

**Background:**

H7 subtype avian influenza has caused great concern in the global poultry industry and public health. The conventional serological subtype-specific diagnostics is implemented by hemagglutination inhibition (HI) assay despite lengthy operation time. In this study, an efficient, rapid and high-throughput competitive enzyme-linked immunosorbent assay (cELISA) was developed for detection of antibodies against H7 avian influenza virus (AIV) based on a novel monoclonal antibody specific to the hemagglutinin (HA) protein of H7 AIV.

**Results:**

The reaction parameters including antigen coating concentration, monoclonal antibody concentration and serum dilution ratio were optimized for H7 antibody detection. The specificity of the cELISA was tested using antisera against H1 ~ H9, H11 ~ H14 AIVs and other avian viruses. The selected cut-off values of inhibition rates for chicken, duck and peacock sera were 30.11, 26.85 and 45.66% by receiver-operating characteristic (ROC) curve analysis, respectively. With HI test as the reference method, the minimum detection limits for chicken, duck and peacock positive serum reached 2^0^, 2^1^ and 2^− 1^ HI titer, respectively. Compared to HI test, the diagnostic accuracy reached 100, 98.6, and 99.3% for chicken, duck and peacock by testing a total of 400 clinical serum samples, respectively.

**Conclusions:**

In summary, the cELISA assay developed in this study provided a reliable, specific, sensitive and species-independent serological technique for rapid detection of H7 antibody, which was applicable for large-scale serological surveillance and vaccination efficacy evaluation programs.

## Background

Avian influenza, a highly contagious respiratory viral disease caused by the avian influenza virus (AIV), continues to impair the domestic poultry and human public health with enormous economic losses alarmingly worldwide [1–3]. AIV, whose genome consists of eight negative sense single-stranded RNA segments that encode at least 11 proteins, belongs to the genus Influenza virus A of the family *Orthomyxoviridae* [4]. According to the antigenic divergence of hemagglutinin (HA) and neuraminidase (NA) surface glycoproteins, AIV comprises 16 HA subtypes and 9 NA subtypes. Influenza A viruses isolated from avian species fall into two pathotypes on the basis of their virulence in chickens: low pathogenicity avian influenza virus (LPAIV) and highly pathogenic avian influenza virus (HPAIV). Among the 16 HA subtypes of AIV, H7 subtype is one of two HA subtypes capable of mutating into HPAIV after transmission to domestic poultry [5]. The HA surface glycoproteins of HPAIV possess multiple basic amino acids at the cleavage site identified by ubiquitous proteases present in a wide range of host cells, allowing for lethal systemic infection in poultry [6].

Phylogenetically, H7 AIV with HA gene compatible with all nine NA subtype genes (N1–N9) is divided into two distinct genetic lineages, North American or Eurasian [7]. So far, infections with H7 AIV have been documented in wild birds, domestic poultry and mammals including humans. Over the past few decades, ongoing outbreaks in poultry caused by HPAIV and LPAIV of the H7N1, H7N2, H7N3, H7N4, H7N6, H7N7 and H7N9 subtypes in both lineages have led to millions of birds’ depopulation [7]. Remarkably, the diversity in geographic distribution of countries affected by the H7 subtype in poultry solidly manifests the global threats towards poultry industry posed by H7 AIV [8, 9]. More seriously, viruses within both American and Eurasian lineages including H7N2, H7N3, H7N7 and H7N9 have turned out to cross the species barrier to cause human infection [10]. The most recent five waves of H7N9 epidemic in China since 2013 further prompt global concerns about an increasing H7 pandemic potential [11]. Therefore, constant vigilance and continuous intensive surveillance are required to minimize the risk of domestic poultry and human infection with the H7 AIV.

As the prerequisite for epidemiologic surveillance studies as well as evaluation of vaccine immunogenicity, serological investigations to detect specific H7 antibodies in poultry are of instructive importance. Classical laboratory serologic tools used for measuring anti-AIV antibodies are agar gel immunodiffusion (AGID), hemagglutination inhibition (HI) test and virus neutralization (VN) test. The AGID test is a simple and economical assay for detection of antibodies to influenza A virus group-specific antigens, namely the ribonucleoprotein and matrix proteins; however, it is also time consuming and not suitable for large-scale clinical specimens screening [12]. HI assay remains the official subtype-specific test for influenza serologic differential diagnosis, but the downsides of this approach include the needs for antigen titration, red blood cells standardization, non-specific inhibitors removal and long processing time for test results [13]. The VN test is recommended for the identification of HPAIV with limited values in rapid and high-throughput diagnostics for the use of live infectious viruses [13]. In comparison to the aforementioned serologic methods, enzyme-linked immunosorbent assay (ELISA) technique is widely accepted as an essential methodology superior in throughput, speed and accuracy for early diagnosis [14]. Among different types of ELISA tests, indirect ELISA is considered as a valuable and inexpensive test routinely used for the detection of antibodies to AIV in birds. However, this technique is not feasible when screening samples from a wide range of bird species. As an alternative, competitive ELISA (cELISA) format, also called epitope-blocking ELISA can achieve this purpose with no need of species-specific enzyme-conjugated antibodies [15].

To successfully construct a qualified cELISA immunoassay, a highly specific monoclonal antibody (mAb) is necessary to recognize a broadly conserved and dominant antigenic epitope throughout H7 strains which can consistently induce antibody response in infected or vaccinated hosts. Notably, HA1 subunit of HA molecule, as the major surface glycoprotein, contains the immunodominant antigenic sites to elicit virus-neutralizing antibodies in birds, rendering it a vital target for mAb production [16]. Yao Lu et al. successfully generated high-affinity mAbs directed against a dominant conserved epitope of recombinant H7-HA1 protein as described previously [17]. In this study, a cELISA immunoassay based on mAb 2F8 to measure serum antibody responses against H7 strains from different susceptible avian species was developed with high specificity, sensitivity and low variability.

## Methods

### Viruses, cells, and animals

The H7 AIV used in the study was a low-pathogenic strain constructed with the internal gene fragments of A/Puerto Rico/8/34 (H1N1) as the backbone through reverse genetics. H7 positive hybridoma cell line 2F8 was cultured in RPMI-1640 medium supplemented with 10% foetal calf serum (Gibco-BRL, USA) at 37 °C with 5% CO_2_. A total of nine six-week-old female BALB/c mice weighing between 22 and 25 g were purchased from the Sino-British SIPPR/BK Lab Animal Ltd. (Shanghai, China). The mice were fed with standard commercial diet and raised in individually ventilated cages in a clean facility at our laboratory.

### Serum samples

The experimental standard sera were obtained from our laboratory as described previously [18]. A total of thirty-six three-month-old specific-pathogen-free (SPF) chickens were purchased from Shennong Company in Zhejiang province of China. They were randomly divided into 18 groups and reared separately in SPF isolators to prepare experimental standard sera. The inactivated AIV (H1N1, H2N2, H3N8, H4N6, H5N1, H6N5, H7N3, H8N4, H9N2, H11N9, H12N5, H13N6 and H14N5), Newcastle disease virus (NDV), avian infectious bronchitis virus (IBV), infectious bursal disease virus (IBDV) and avian leukosis virus-J subgroup (ALV-J) were emulsified in complete Freund’s adjuvant (1,1) and injected subcutaneously to the SPF chickens, respectively. The above virus strains were preserved at − 80 °C at the author’s laboratory. Another group of two SPF chickens were injected with phosphate buffered saline (PBS) as a negative control. After 2 weeks, a second immunization was administered with the above inactivated viruses emulsified 1:1 in incomplete Freund’s adjuvant. A booster was administered at a two-week interval. Standard monospecific antisera were prepared from the blood collected 10 days after the booster immunization (Table 1). Standard negative sera were harvested from SPF chickens prior to immunization. After the blood was collected from leg veins of SPF chickens, they were euthanized in CO_2_. These experimental sera were separated from whole blood by centrifugation at 2000×g for 10 min after overnight incubation at 4 °C and then stored at − 40 °C until use. They were previously evaluated by HI assays or commercial ELISA kits and then subjected to the cELISA assay.

**Table 1:** The information profile of standard positive chicken antisera

Strain name	Strain description	Highly similar sequences^a^ (≥99%)	HI titer (2^x^)
AIV-H1N1 P2009	H1N1	MH061695.1	10
AIV-H2N2 21103	H2N2	L11134.1	10
AIV-H3N8 11102	H3N8	CY005816.1	8
AIV-H4N6 20411	H4N6	GU052381.1	7
AIV-H5N1 060315	H5N1	JX565019.1	9
AIV-H6N5 20411	H6N5	CY014656.1	9
AIV-H7N3 201369	H7N3	JQ906576.1	6
AIV-H8N4 20413	H8N4	CY014659.1	9
AIV-H9N2 201313	H9N2	KF059279.1	10
AIV-H11N9 21103	H11N9	CY014687.1	8
AIV-H12N5 11103	H12N5	GU052216.1	5
AIV-H13N6 11103	H13N6	CY014694.1	8
H14-A1131028	H14N5	KF986854.1	10
NDV (Lasota)	NDV	DQ195265.1	9
IBV-J (F8)050309	IBV	FJ849834.1	–
IBDV-NB(F7)	IBDV	AY319768.2	–
ALV-J	ALV-J	KM655820.1	–

^a^Accession number from the GenBank databases

In this study, a total of 400 clinical serum samples from chicken, duck and peacock were obtained. The 260 chicken and duck clinical serum samples used in this study were collected from poultry farms of Jiangsu province in China and categorized into positive or negative sera by HI assay. The 140 peacock clinical serum samples were gathered from Nanjing Hongshan Forest Zoo, of which the 100 positive peacock clinical sera verified by HI assay were collected sequentially 1 month after the immunization of reassortant AIV (H5 + H7) trivalent vaccine, and 40 negative peacock clinical sera were collected before immunization.

### Identity and preparation of antigen

Each 10-day-old SPF embryonated chicken egg was inoculated via the allantoic cavity with the H7 AIV diluted in 100 μL 0.01 M sterile PBS. During the incubation period at 37 °C for 2–3 days, eggs were monitored twice daily for embryo mortality. Embryos which died within 24 h were discarded. After eggs were chilled at 4 °C overnight, allantoic fluids confirmed by hemagglutination (HA) test were aseptically harvested and combined. The allantoic fluids of H7 viruses were further inactivated by administration of β-propiolactone diluted at a proportion of 1:2000 with PBS at 4 °C for 12 h. An inactivated status was detected by antigen challenge in 10-days-old SPF eggs for 3 days to check for live viruses by HA test. The validated inactivated virus suspensions were further clarified by centrifugation at 12,000×g at 4 °C for 10 min. Virus supernatants were collected and pelleted by ultracentrifugation at 110,000×g at 4 °C for 1.5 h and subsequently the virus precipitates were suspended by 3–6 mL PBS. Thereafter, the precipitated viruses were ultracentrifuged at 160,000×g at 4 °C for 3 h by using the sucrose gradient centrifugation to remove impurities of different densities. Finally, the purified viruses underwent ultracentrifugation at 160,000×g at 4 °C for 2.5 h to remove sucrose followed by suspension with 2 mL PBS. The successfully purified viruses were evaluated and stored at − 80 °C. All viral manipulations were performed under appropriate biosafety level 2 laboratory conditions.

### Preparation of anti-H7-HA1 mAb

The mAb 2F8 directed against recombinant H7-HA1 protein was produced as described previously [17]. In brief, six-week-old female BALB/c mice were first injected at multiple sites subcutaneously with 50 μg of recombinant H7-HA1 protein mixed with an equal volume of Freund’s complete adjuvant, and then injected intraperitoneally with 50 μg of recombinant H7-HA1 protein mixed with an equal volume of Freund’s incomplete adjuvant for two times at 14 and 28 days after the first injection. Three days after the final injection with 100 μg of the protein, spleen cells from the immunized mice were fused with myeloma cells (sp2/0), and the positive hybridoma were screened by indirect ELISA results against recombinant H7-HA1 protein. The selected clones were further subcloned four times by limiting dilution to obtain stable monoclone, and confirmed by western blot assay, immunofluorescence assay, HI assay, blocking assay and neutralization assay.

Each mouse received an intraperitoneal inoculation of 0.5 mL liquid paraffin. Following 7 days, hybridoma cells were diluted in serum-free RPMI-1640 medium. The mAb 2F8 ascites were prepared by injecting intraperitoneally 5 × 10^6^ diluted positive hybridoma cells into each paraffin-primed six-week-old BALB/c mice. Following a further 7 days, mouse ascites production was monitored daily. Ascites were considered to have generated when obvious abdominal swelling was observed and skin tension was palpable. At the end of the experiment, all the mice were euthanized via CO_2_ inhalation. Subsequently, the mAb 2F8 was purified from ascites via caprylic acid-ammonium sulfate precipitation followed by HiTrap Protein G affinity chromatography (Amersham, Sweden) according to the manufacturer’s instructions.

### Establishment of the cELISA immunoassay

**Fig. 1:**
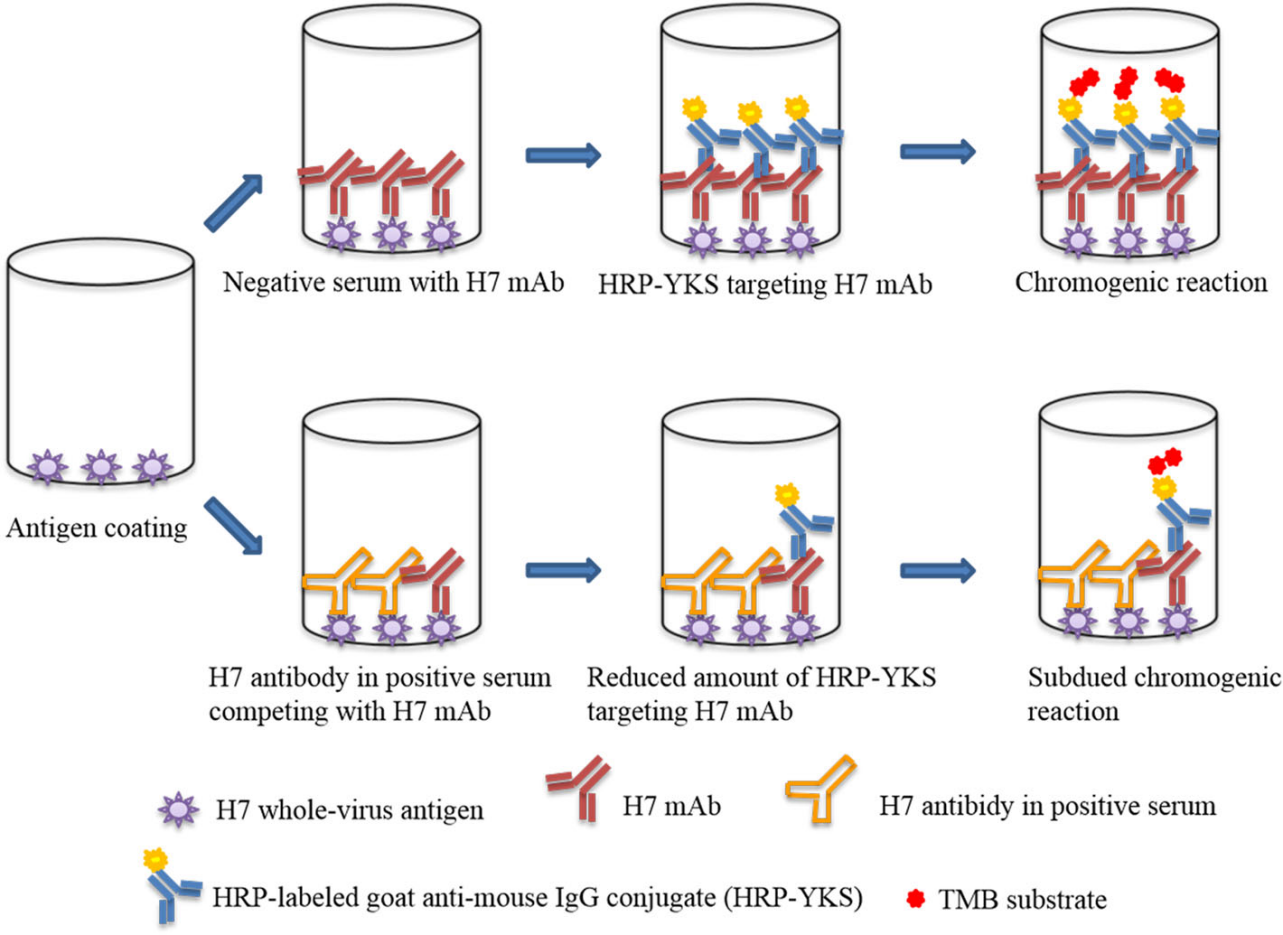
Schematic illustration of the cELISA immunoassay for the detection of H7 antibody. A 96-well microtiter plate coated with purified inactivated H7 whole-virus antigen is prepared. The mixture of primary competitive mAb and unknown serum sample is diluted to optimal concentration and added to the antigen coated plate. After an incubation period of 1 h, the plate is washed to remove unbound antibody and a secondary antibody labeled with horseradish peroxidase specific for targeting the competitive mAb is added. After another incubation of 30 min, the plate is washed again to remove unbound secondary antibody and then the substrate is added. If the serum sample is negative, the enzyme on secondary antibody which binds specifically the murine competitive mAb linked on the antigen-coated plate will catalyze the substrate and result in a color change. In contrast, the antibody in positive serum sample will compete with competitive mAb for capturing the same epitope of coated antigen. Subdued color change will be observed due to the species-specific property of the secondary antibody. The color intensity resulting from antigen bound mAb is inversely proportional to the amount of epitope-specific antibody present in test serum

The cELISA assay runs according to the workflow illustrated in Fig. 1. Each well of 96-well microtiter plates (JET BIOFIL, China) was coated overnight at 4 °C with 100 μL coating buffer (0.05 M carbonate/bicarbonate buffer, pH = 9.6) containing the purified H7 inactivated whole virus particles at working concentration. After five rapid washes with 300 μL PBS buffer containing 0.05% Tween-20 (PBST), plates were then blocked with 300 μL blocking solution (PBS buffer containing 5% skimmed milk) at 37 °C for 1 h. After rinsing five times with PBST, equal volumes of unknown serum samples and competitive mAb diluted to working concentration with blocking buffer were mixed and incubated simultaneously at 37 °C for 1 h. Wells added with competitive mAb mixed with standard monospecific antisera, negative sera or no serum served as positive, negative or blank control respectively. To provide results statistically valid, each serum sample was tested in triplicate. After washing steps for five times, 100 μL horseradish peroxidase-labeled goat anti-mouse IgG conjugate diluted to an optimal dilution was placed in each well at 37 °C for 30 min, followed by the same washing steps as described above. After 100 μL tetramethylbenzidine (TMB) substrate was added to each well followed by incubation in the dark at room temperature for 5 min, 50 μL stop solution (2 M sulfuric acid) per well was added to terminate the colorimetric reaction and optical density at 450 nm (OD_450_) were measured by an automated multimode reader. Results were interpreted as the percentage of inhibition (PI) calculated according to the following formula: % inhibition = ((OD_450_ of mAb-OD_450_ of serum sample)/OD_450_ of mAb) × 100%.

To standardize the assay, the optimal concentrations of inactivated antigen and competitive mAb were determined by means of checkerboard titration. The coating antigen concentrations in the range of 2, 4, 6 and 8 μg/mL and the two-fold serial dilutions of the purified mAb ascites fluids from 0.3125 μg/mL to 1.25 μg/mL were tested respectively. The optimal parameters were determined by the highest PI value and then used in the following validation assays. Subsequently, a receiver-operating characteristic (ROC) curve analysis was carried out to determine the cut-off points and assess the discriminating performance of the established cELISA assay for H7 antibody detection with HI assay as the gold standard test.

### Specificity and sensitivity for H7 antibody detection

A panel of standard monospecific antisera produced against other non-H7 AIVs, NDV, IBV, IBDV and ALV-J were used to assess the specificity of the cELISA assay. In order to test the sensitivity of established cELISA assay to detect H7 antibodies, two-fold serial dilutions of chicken, duck and peacock hyperimmune sera against H7 subtype starting from one-quarter were subjected to the cELISA by comparison to homologous HI assay. The chicken hyperimmune sera used for sensitivity test were selected from both experimental standard sera and field sera samples confirmed by HI assay; the duck and peacock hyperimmune sera were selected from field sera samples confirmed by HI assay.

### Repeatability test

In order to analyze the reproducibility and reliability of the cELISA immunoassay, the same positive and negative chicken, duck and peacock sera verified by HI assay were tested by the same batch and different batches of constructed ELISA plates. The chicken sera were selected from prepared experimental standard sera and field sera samples verified by HI assay; the duck and peacock sera were selected from clinical sera samples verified by HI assay. The mean PI values, standard deviation (SD) and coefficient of variation (CV) were calculated to measure the uniformity within and between batches of the cELISA assay. The CV is defined as the ratio of the SD to the mean.

### Clinical applicability study

For evaluation of clinical application performance, a total of 400 field serum samples from chicken, duck and peacock were tested for the presence of H7 antibody by the cELISA assay and homologous HI test in parallel. Subsequently, The diagnostic sensitivity, specificity and accuracy of the cELISA assay compared to HI test were calculated by the following formulas: Sensitivity = True positive/(True positive+False negative) × 100%, Specificity = True negative/(False positive+True negative) × 100% and Accuracy = (True positive+True negative)/total number of serum samples tested× 100%.

### HI assay

The HI assay was performed in microtiter plates as described previously with 1% chicken red blood cells and 8 hemagglutinin units (8 HAU) of virus [19]. One HA unit is represented by the highest dilution of virus giving complete hemagglutination. The HI titer is expressed as the highest dilution of serum resulting in complete inhibition of 8 HAU of virus. The HI titer is regarded as being positive if there is inhibition at a serum dilution of 1/16 or more against 8 HAU of virus. In order to validate the results of the established cELISA for H7 antibody detection, all sera were tested by both HI assay and the cELISA for H7 antibody detection in parallel.

### Statistical analysis

The area under ROC curves (AUC) and optimal cut-off points of the cELISA assay were determined with HI assay as the gold standard method by using Graphpad Prism Software Version 5.0. The AUC value describes an overall summary statistic of diagnostic accuracy which can distinguish between non-informative (AUC = 0.5), less accurate (0.5 < AUC ≤ 0.7), moderately accurate (0.7 < AUC ≤ 0.9), highly accurate (0.9 < AUC < 1) and perfect tests (AUC = 1). The optimal cutoff points were ascertained as the serum antibody titers corresponding to the PI values at which both specificity and sensitivity of the assay were maximized.

The correlation between the cELISA and HI test was interpreted by the Pearson correlation coefficient (r) which varies between − 1 and + 1. Zero implies there is no correlation while 1 implies a perfect correlation. The strength of the correlation rises from 0 to + 1, and from 0 to − 1. The *p*-value manifests the probability that the strength of correlation may occur by chance.

The strength of agreement between the cELISA and homologous HI test for field serum sample testing was assessed by Cohen’s κ value through IBM SPSS Statistics 23. The κ values are interpreted in line with the criteria given by Landis and Koch [20]. (κ ≤ 0.00 was designated as poor agreement, 0.00 < κ ≤ 0.20 slight agreement, 0.21 < κ ≤ 0.40 fair agreement, 0.41 < κ ≤ 0.60 moderate agreement, 0.61 < κ ≤ 0.80 substantial agreement, and 0.81 < κ < 1.00 almost perfect agreement.)

## Results

### Development of the H7 cELISA

The mAb 2F8 is an IgG2a isotype with kappa light chains. The concentration for purified ascites of 2F8 was 2 mg/mL. The purified ascites of mAb 2F8 served as the competitive mAb for assay establishment.

**Fig. 2:**
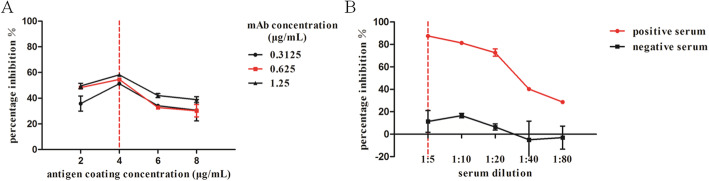
Optimization of test parameters for the cELISA immunoassay. **a** The coating antigen concentrations of 2, 4, 6 and 8 μg/mL and 2-fold serial dilutions of the 2F8 purified ascites varied from 0.3125 to 1.25 μg/mL were tested respectively by checkerboard titration. **b** After the optimal antigen and competitive antibody concentrations were determined, the optimal dilution ratio for test sera was determined by 2-fold serial dilution from 1:5 to 1:80. The combination that gave the highest PI values was determined to be 4 μg/mL antigen, 0.625 μg/mL mAb and 1:5 dilution ratio of test serum. The red vertical dotted lines indicate the optimal working condition

By way of checkerboard titration analysis, the optimal concentration of coated antigen was fixed on 4 μg/mL and the optimal concentration of 2F8 competitive mAb was fixed on 0.625 μg/mL (Fig. 2). The 1/5 dilution of test sera was selected considering the highest PI values and widest window for detection of positive and negative serum samples (Fig. 2). The most suitable dilution ratio for horseradish peroxidase-labeled goat anti-mouse IgG conjugate was 1:5000. These optimized parameters were applied for further validation of the cELISA.

Comparison was conducted to evaluate the effectiveness for H7 antibody detection of these two reaction patterns, including simultaneous incubation and step-by-step incubation of test sera and competitive mAb. It was indicated that the cELISA using simultaneous incubation of test sera and competitive mAb showed best diagnostic performance and reduced assay duration and was then selected for further validation.

**Fig. 3:**
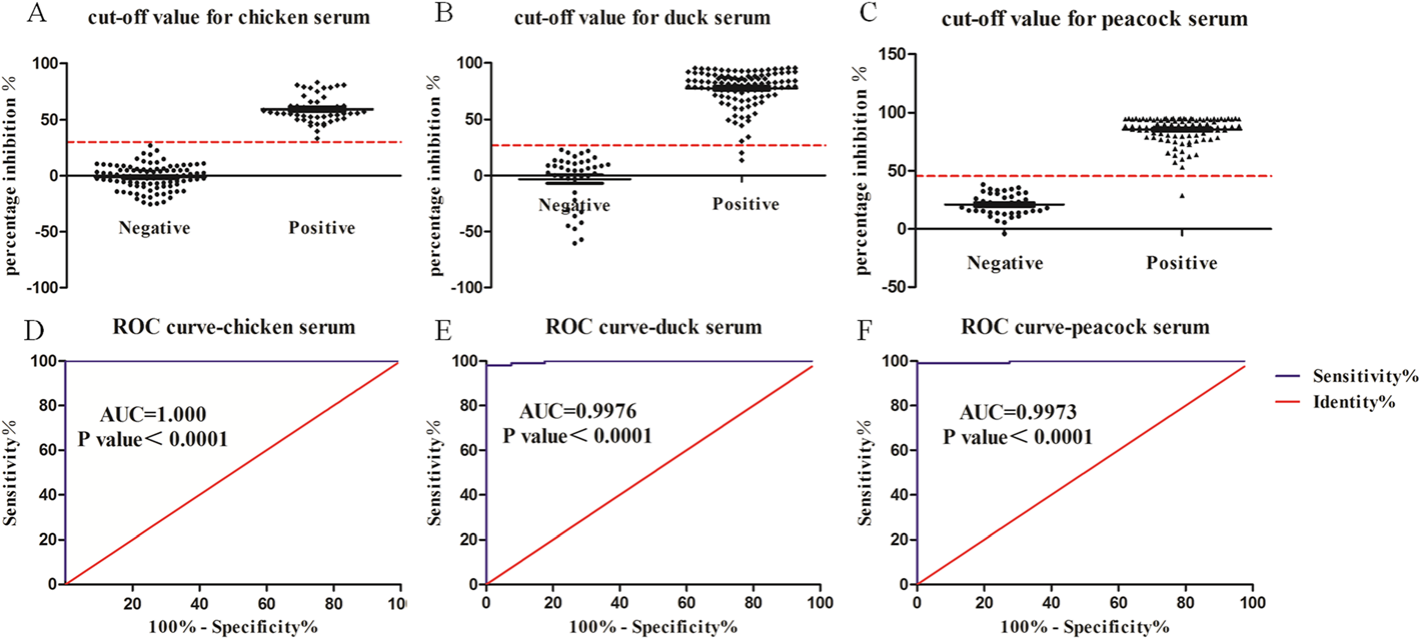
Interactive dot diagram for detection of H7 antibodies in chicken (**a**), duck (**b**) and peacock (**c**) sera and ROC curves for detection of H7 antibodies in chicken (**d**), duck (**e**) and peacock (**f**) sera used to set the cut-off values for the cELISA immunoassay. Each dot represents one serum sample. The horizontal dotted lines indicate the optimal cut off levels with the best sensitivity and specificity. AUC represents area under the curve

A panel of 435 chicken, duck and peacock experimental and field sera confirmed as positive or negative by HI assay were used for construction of ROC curves. Positive sera included samples which cover the range of antibody titers from high to low levels. The AUC values suggested that the cELISA assay for H7 antibody detection was highly accurate (AUC = 1, 0.9976 and 0.9973 for differentiation of chicken, duck and peacock positive and negative sera respectively). ROC analysis for the cELISA was performed over a range of possible cut-off points (Table 2). The optimal cut-off values to define positive and negative test outcomes were determined to be 30.11, 26.85 and 45.66% for chicken, duck and peacock test sera respectively, representing the optimal balance of all 100% diagnostic specificity and sensitivity for chicken sera, 100% diagnostic specificity and 98.10% sensitivity for duck sera, 100% diagnostic specificity and 99% sensitivity for peacock sera (Fig. 3).

**Table 2:** Evaluation of the cELISA immunoassay with selected cut-off values

Species	Cut-off values (%)	Sensitivity (%)	Specificity (%)
Chicken	24.77	100.0	99.00
	30.11	100.0	100.0
	36.50	98.00	100.0
	42.25	96.00	100.0
Duck	22.39	98.10	97.50
	26.85	98.10	100.0
	32.63	97.14	100.0
	39.50	96.19	100.0
Peacock	36.88	99.00	97.50
	45.66	99.00	100.0
	55.21	98.00	100.0
	58.67	97.00	100.0

### Specificity and sensitivity of the H7 cELISA

**Fig. 4:**
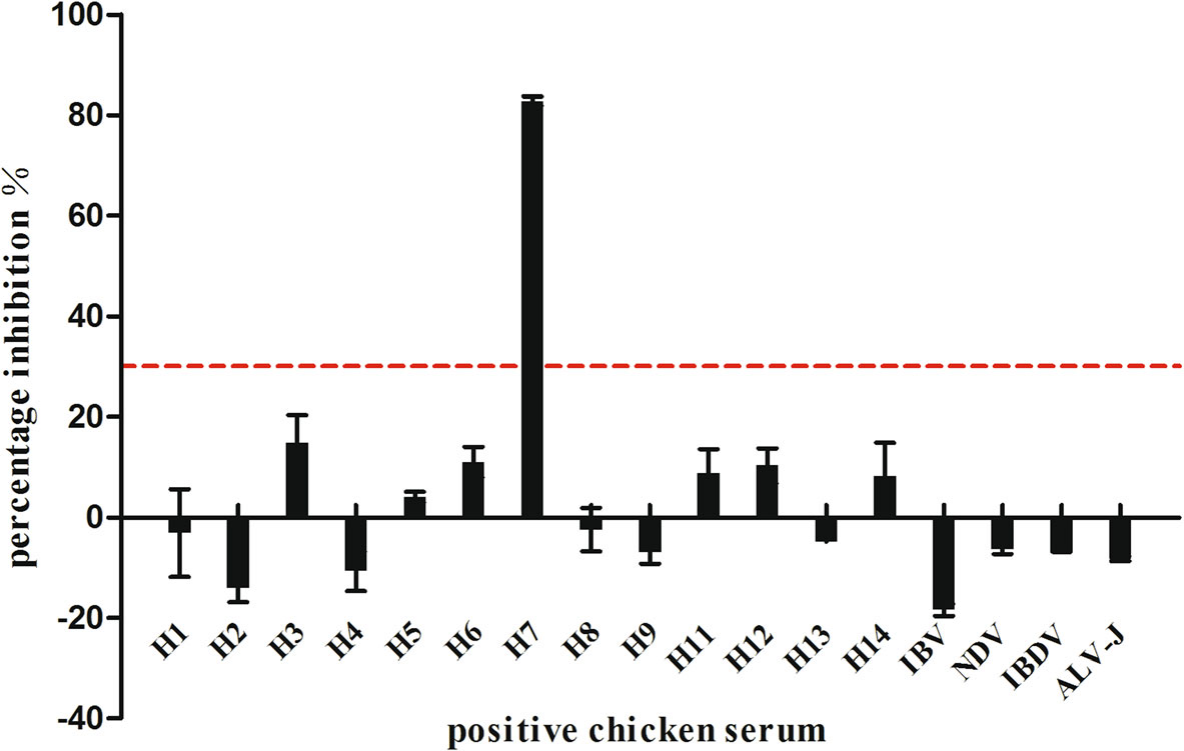
The cross-reactivity of the competitive ELLISA immunoassay with standard chicken positive antisera against different HA subtypes of AIV and other avian viruses. The horizontal dotted lines indicate the cut-off value of 30.11% for chicken sera. The arithmetic mean of PI values for standard positive sera against AIV of H1-H9 and H11-H14 subtypes, NDV, IBV, IBDV and ALV-J were much lower than 30.11% except for the H7 positive serum

As was shown in Fig. 4, the H7 monospecific standard chicken serum gave over 80% inhibition percentage while standard sera containing antibodies directed against other subtypes and other avian viruses gave inhibition percentage from − 14.72 to 20.27%, significantly lower than the detection threshold. The H7 cELISA assay could specifically detect H7 antibodies against H7 AIV in positive sera, and had no serological cross-reactivity with sera against other non-H7 AIVs, NDV, IBV, IBDV and ALV-J.

**Fig. 5:**
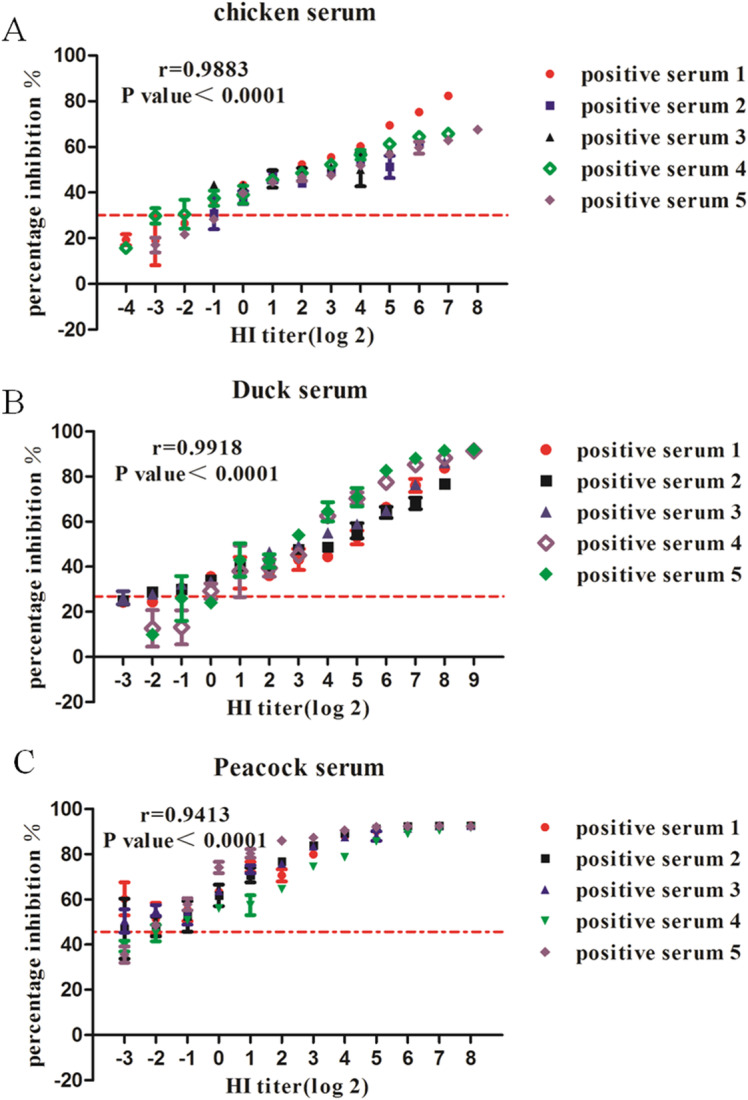
The sensitivity of the H7 cELISA assay for the detection of H7 antibody and the correlation between H7 cELISA and HI assay. A panel of H7 strong positive chicken (**a**), duck (**b**) and peacock (**c**) sera were twofold diluted and subjected to the cELISA and HI assay for H7 antibody detection. The horizontal dotted lines indicate the cut off value and r represents Pearson correlation coefficient

In this study, a pool of two-fold serially diluted strong positive sera from chicken, duck and peacock were subjected to both the cELISA and homologous HI assay for assessment of analytical sensitivity for the cELISA immunoassay and their correlation. As illustrated in Fig. 5, a strong correlation was observed between PI values and HI titers on a logarithmic scale with a very high statistical significance (*p* < 0.0001) according to r values reported to be 0.9883, 0.9918 and 0.9413 for chicken, duck and peacock test sera, respectively (95% confidence interval). In the light of determined optimal cut-off values to discriminate between positive and negative sera, the analytical sensitivity for the cELISA was reported down to 2^0^, 2^1^ and 2^− 1^ HI titer for chicken, duck and peacock test sera.

### Stability of the H7 cELISA immunoassay

The evaluation of the reproducibility was carried out within and between runs for analysis of chicken, duck and peacock sera confirmed by HI assay respectively. Through statistical analysis, the *intra*-batch CVs of the 15 tested sera ranged from 0.19 to 6.17%, whereas the *inter*-batch CVs ranged from 2.94 to 15.15% (Table 3). These results demonstrated that the cELISA immunoassay were reproducible with low and acceptable variation.

**Table 3:** *Intra* and *Inter*-assay repeatability of the cELISA immunoassay

Species	Serum samples	HI titer	Inhibition percentage					
			*Intra*-assay			*Inter*-assay		
			Mean (%)	SD	CV (%)	Mean (%)	SD	CV (%)
Chicken	Positive serum 1	2^6^	77.30	0.014	1.92	76.34	0.052	6.91
	Positive serum 2	2^8^	78.34	0.003	0.35	70.07	0.095	13.62
	Positive serum 3	2^9^	81.50	0.005	0.67	85.76	0.049	5.74
	Negative serum 1	–	29.01	0.002	0.82	29.76	0.013	4.43
	Negative serum 2	–	14.89	0.009	6.17	19.50	0.027	14.04
Duck	Positive serum 1	2^10^	89.69	0.011	1.22	84.20	0.064	7.56
	Positive serum 2	2^10^	88.75	0.007	0.81	81.50	0.091	11.12
	Positive serum 3	2^10^	86.72	0.017	1.91	81.73	0.059	7.18
	Negative serum 1	–	13.00	0.002	1.57	13.21	0.004	2.94
	Negative serum 2	–	22.39	0.007	3.27	20.61	0.031	15.15
Peacock	Positive serum 1	2^9^	85.46	0.014	1.69	80.66	0.056	6.96
	Positive serum 2	2^9^	85.07	0.024	2.85	89.63	0.054	6.07
	Positive serum 3	2^9^	93.99	0.002	0.19	90.13	0.045	4.95
	Negative serum 1	–	15.94	0.001	0.20	15.66	0.014	8.74
	Negative serum 2	–	11.12	0.002	1.66	11.87	0.013	11.04

### Diagnostic performance in field samples

To test the applicability in clinical use, a total of 400 field serum specimens from chicken, duck and peacocks were screened for the existence of H7 antibodies by using cELISA and HI assay (Table 4). It was shown that a total of 245 out of the 400 samples tested positive, and 155 samples tested negative according to the HI assay while a total of 242 positive samples and 158 negative samples were detected by the cELISA. Overall, the positive rates for HI assay and the cELISA were calculated as 61.3 and 60.5% respectively. For chicken test sera, the cELISA was found to be 100% specific and 100% sensitive relative to HI assay; for duck test sera, the cELISA was found to be 100% specific and 98.1% sensitive relative to HI assay; for peacock test sera, the cELISA was found to be 100% specific and 99% sensitive relative to HI assay. The diagnostic accuracy of the cELISA relative to HI assay for chicken, duck and peacock test sera were reported to be 100, 98.6 and 99.3% respectively. The agreement between the cELISA and HI assay was assessed by Cohen’s κ analysis. According to the reported κ values (κ = 1.000 for chicken sera; κ = 0.966 for duck sera; κ = 0.983 for peacock sera), an almost perfect agreement was observed between the two methods for the three species.

**Table 4:** Comparison study between the cELISA immunoassay and HI assay for the detection of H7 antibody in chicken, duck and peacock field serum samples

Target	Chicken serum		Duck serum		Peacock serum	
	H7 cELISA	HI test	H7 cELISA	HI test	H7 cELISA	HI test
Positive	40	40	103	105	99	100
Negative	75	75	42	40	41	40
Positive rates	34.8%	34.8%	71.0%	72.4%	70.7%	71.4%
Sensitivity ^a^(TP/(TP + FN))	100%		98.1%		99%	
Specificity ^b^(TN/(TN + FP))	100%		100%		100%	
Accuracy ^c^((TP + TN)/Total)	100%		98.6%		99.3%	

^a^ *TP* true positive, *FN* false negative; sensitivity = (TP/(TP + FN)) × 100%

^b^ *TN* true negative, *FP* false positive; specificity = (TN/(TN+ FP)) × 100%

^c^ Total, total number of serum samples; Accuracy = ((TP + TN)/Total) × 100%

## Discussion

The continual prevalence of H7 AIV in poultry constitutes an enormous challenge towards the poultry industry and human public health. Effective prevention and control of H7 outbreaks require active global serological surveillance in poultry. For early detection and control of subclinical H7 infection, extensive serological surveillance programs in poultry have been carried out by the European Union [21]. Moreover, full-scale vaccination campaigns have been enforced throughout China to prevent H7 outbreaks in poultry since 2017. With the increase of sampling for sero-surveillance and vaccination efficacy studies, it is urgent to develop novel rapid H7 subtype-specific serological assay for antibody screening. Currently, the cELISA techniques contribute to the rapid high-throughput detection of subtype-specific AIV antibodies in poultry flocks. This study aimed to develop and validate a cELISA immunoassay for H7 antibody early detection in sera from different avian species after viral infection and vaccination.

In this study, the effect of purified H7 whole virus particles or histidine-tagged recombinant H7-HA1 protein expressed in a prokaryotic system as the coating antigen was investigated. And the finding was that the same standard positive antisera against H7 subtype exhibited a higher PI value with purified H7 whole virion as the coating antigen in comparison to recombinant H7-HA1 protein. Possible explanation for this disparity was that the denatured recombinant H7-HA1 protein differed from the native structure of the corresponding HA1 domain of H7 AIV.

The advantages of using mAbs for detecting AIV include high specificity, sensitivity and unlimited provision of a standardized reagent. mAbs against the HA protein of AIV served as a useful tool to distinguish its subtypes in previous studies [22–24]. In the present paper, the cELISA immunoassay for H7 antibody detection was developed using an anti-H7-HA1 mAb targeting the epitope located in the depression under antigenic site E of H7-HA1 subunit [17]. Through alignment of epitope sequences, the epitope recognized by mAb 2F8 was found to be highly conserved in H7 strains of Eurasian lineage while not found in any other subtypes of AIV [17]. At present, the commercial H5/H7 trivalent vaccine used in China was developed by using inactivated whole virus containing major HA antigen, thus the vaccination induced the same type of antibodies in poultry as those infected naturally. Given that the established cELISA assay detected specific antibodies against the HA protein of H7 AIV, it will be useful for sero-surveillance investigation but not suitable for outbreak diagnosis.

Conventionally, the Gaussian distribution approach is commonly used to identify cut-off values for diagnostic tests. For this approach, a cut-off value is defined as the mean plus two standard deviations (2SD) of the negative reference samples, which ensured a diagnostic specificity of 97.5% without considering the diagnostic sensitivity [25]. In this study, we determined the baseline cut-off values by use of ROC curves analysis which enabled a combined measure of diagnostic sensitivity and specificity. For serological subtyping of the avian influenza virus, HI test is regarded as the gold standard in routine diagnostic practice recommended by OIE. So the ROC curve analysis was built on the classification results of sera by HI assay. For ROC analysis, the AUC determines the inherent predictive ability of the test to discriminate between positive and negative sera. In this study, the developed cELISA assay turned out to be significantly reliable with the threshold PI values of 30.11, 26.85 and 45.66% for chicken, duck and peacock test sera (AUC = 1, 0.9976 and 0.9973, *p* value < 0.0001, satisfactory sensitivity and specificity were achieved). It was shown that the determined cut-off value for peacock sera was higher than that for chicken and duck sera. Such variation among different avian species has been already reported in the literature [26]. The different reactivity of sera across distinct species with the same coating antigen can be explained by the various serum compositions and immune responses in a species-dependent manner.

The cELISA immunoassay format is expected to test sera from diverse hosts without changing any test reagents. To verify the capability of the developed cELISA assay to analyze sera from different hosts, field specimens from a variety of avian species were used for H7 antibody detection, including chicken, duck and peacock. It was shown that the cELISA assay yielded diagnostic results in a high accordance rate with the gold standard HI assay for H7 antibody detection in accordance with the determined cut-off values. Based on this study, the constructed multi-species cELISA assay is of practical use for evaluating vaccination effectiveness of zoo birds other than domestic poultry and waterfowl. The cELISA assay will be further evaluated and applied in other avian species.

The newly developed cELISA immunoassay has been proved to be more effective in terms of sensitivity, efficiency and biosafety. As demonstrated by the results that detection limits of the H7 antibodies for chicken, duck and peacock test sera reached 2^0^, 2^1^ and 2^− 1^ HI titer respectively, the cELISA was superior in analytical sensitivity when compared to HI assay by which a serum with 2^4^ or more than 2^4^ HI titers was diagnosed as being positive. Furthermore, the analytical sensitivity for chicken sera was improved by 16-fold using this novel immunoassay as confirmed by comparison of the previously developed cELISA assay [27]. Although the HI assay is routinely used for subtyping in AIV serologic surveillance, it is laborious and time-consuming for red blood cells preparation, antigen cultures, serum samples serial dilutions and manual reading of results. The pan-subtype cELISA assay can serve as a multi-species AIV serological screening tool yet HI assay is required for further subtyping [28]. In this study, the developed cELISA assay is easy to automate for large-scale monitoring of H7 subtype-specific antibody in sera from various avian species for its simplicity of operation, instrumental reading and easy interpretation of the results. It is worth mentioning that the entire test only takes about 95 min, which is several times faster than the HI test when processing a large number of samples. In addition, the established cELISA assay had a biosafety advantage in comparison to HI assay. The established cELISA assay is easy to be applied in laboratories with low-level biosafety regulations, while HI assay can only be operated in the high-level biosafety laboratories when handling live virus.

Overall, an excellent correlation was observed between the HI test and the novel cELISA immunoassay for the detection of H7 antibody. For the tested panels of field serum samples, diagnostic results for the cELISA assay were highly consistent with homologous HI assay along with 100, 98.6 and 99.3% diagnostic accuracy for chicken, duck and peacock sera. An excellent agreement was observed between HI assay and the novel cELISA immunoassay for clinical diagnostic performance by Cohen’s κ analysis. Considering these advantages, the cELISA assay introduced in this study turned out to be a valid and efficient tool for titration of H7 antibodies in large amounts of chicken, duck or peacock sera.

Both H5 and H7 subtype AIV should be reported to OIE for their high risk of becoming HPAIV after transmission to domestic poultry. Furthermore, their devastating impact on the poultry industry and zoonotic potential underscore the great need to develop rapid and effective serological screening tools for early detection and control of subclinical H5 and H7 infection. Therefore, further studies should be performed to develop cELISA assays for simultaneous detection of antibodies against both H5 and H7 subtype AIV.

## Conclusions

Considering the high agreement with HI assay, the cELISA assay based on anti-H7-HA1 mAb and whole-virus virion described in this paper proved to be an attractive choice for evaluating the specific antibody levels against H7 subtype in serum samples from different avian species of interest. It was amenable to rapid and high throughput screening for H7 antibody with brilliant specificity, sensitivity and reproducibility. In the future, it offers a promising approach for massive epidemiological screening and sero-surveillance of antibody response against H7 subtype.

## Data Availability

The datasets used and/or analyzed during the current study are available from the corresponding author on reasonable request.

## Abbreviations

AGID: Agar gel immunodiffusion
AIV: Avian influenza virus
ALV-J: Avian leukosis virus-J subgroup
AUC: The area under ROC curves
cELISA: Competitive enzyme-linked immunosorbent assay
CV: Coefficient of variation
ELISA: Enzyme-linked immunosorbent assay
HA: Hemagglutinin
HAU: Hemagglutinin units
HI: Hemagglutination inhibition
HPAIV: Highly pathogenic avian influenza virus
IBDV: Infectious bursal disease virus
IBV: Avian infectious bronchitis virus
LPAIV: Low pathogenicity avian influenza virus
mAb: Monoclonal antibody
NA: Neuraminidase
NDV: Newcastle disease virus
VN: Virus neutralization
OD_450_: Optical density at 450 nm
PBS: Phosphate buffered saline
PBST: PBS buffer containing 0.05% Tween-20
PI: Percentage of inhibition
ROC: Receiver-operating characteristic
SD: Standard deviation
SPF: Specific-pathogen-free
TMB: Tetramethylbenzidine
